# Analysis of College Students’ Entrepreneurship Education and Entrepreneurial Psychological Quality From the Perspective of Ideological and Political Education

**DOI:** 10.3389/fpsyg.2022.739353

**Published:** 2022-04-01

**Authors:** Yuqian Jin

**Affiliations:** Department of Faculty Affairs & Faculty Development, School of Marxism, Northwestern Polytechnical University, Xi’an, China

**Keywords:** ideological and political education, college students, entrepreneurship education, innovative thinking, psychological quality

## Abstract

The purpose is to meet the needs of social development, encourage college students’ employability, and alleviate social pressure. First, the domestic and international employment situation is discussed along with the derivation of entrepreneurship education. Second, ideological and political education is summarized, and accordingly, its internal advantages and external conditions are explained. Then, the current situation and existing problems of entrepreneurship education in Chinese higher education institutions are analyzed from the perspectives of entrepreneurship ideology, entrepreneurship thinking, and entrepreneurship ability. Consequently, a proposal is put forward to integrate entrepreneurship education into ideological and political education to promote the coordinated development of the multidisciplinary course. Finally, the Questionnaire Survey (QS) is designed on the relationship between college students’ entrepreneurship education and ideological and political education, and the QS is conducted from three aspects: college students’ entrepreneurship views, suggestions, and cognition of the ideological and political education. The QS results show that less than 10% of students are very interested in entrepreneurship education, and most students believe that entrepreneurship education plays a very limited role in their growth. Additionally, classroom teaching accounts for the most in entrepreneurship education, while extracurricular practice accounts for only 15%. In terms of teaching content, 80% of students believe that teachers of professional courses have made outstanding contributions to teaching results, but the number of teachers of professional courses is the least among all teachers. Further, the satisfaction of entrepreneurship education among college students is analyzed through the QS by issuing to three local higher education institutions, finding that the college students’ understanding of entrepreneurship education is incomplete. The advantages of ideological and political education should be fully exerted to improve the recognition of entrepreneurship education, and entrepreneurship education should be integrated into daily teaching content. In this way, entrepreneurship education and ideological and political education can be fused, and entrepreneurship education can promote students’ entrepreneurial ability and entrepreneurial thinking.

## Introduction

The number of college graduates in China has been on a constant rise in recent years, pressing on greater employment stress. Many college students cannot receive a job offer after graduation, and they are worried about not finding a satisfactory job. To alleviate unemployment for college students, college students’ entrepreneurship has been greatly encouraged by the government through many favorable policy packages, only to find a minute effect. Many college students choose entrepreneurship only to temporarily get away from layout dilemmas without really understanding the importance of entrepreneurship (Chen, 2021). The last decades of the 20th century have witnessed the popularization of entrepreneurship education in American higher education institutions. With the increasing employment pressure, entrepreneurship education is becoming extremely important in the education system of higher education institutions. Many well-known universities in foreign countries develop entrepreneurship education for students from enrollment to graduation (Zhao et al., 2020), with diversified out-of-class activities. These foreign schools invite their entrepreneurial alumni to lecture on the current employment situation to help students better evaluate and consider entrepreneurship. Comparatively, most domestic universities only offer entrepreneurship education to senior students (Yan, 2021). Due to limited theoretical cultivation and lack of practical activities, students can hardly grasp entrepreneurship, not even to mention the number of successful student entrepreneurs (Obschonka and Stuetzer, 2017).

The accelerated economic globalization has brought severe challenges to the domestic talents market. The importance of entrepreneurship has been deeply realized by the Chinese government, and a series of policy packages to strengthen entrepreneurship education have been released. Accordingly, domestic higher education institutions should pay more attention to entrepreneurship education for college students and should establish an effective evaluation system for comprehensive entrepreneurship ability, such as students’ psychological quality and professional skills (Huq and Gilbert, 2017). Given these efforts, domestic entrepreneurship education has gained considerable signs of progress, yet the educational quality and effect are indistinct compared with international standards. Students’ entrepreneurial enthusiasm has not been fully stimulated, and their entrepreneurial ability needs further improvement. The key to these problems lies in a sound and all-around cultivation of students’ ideological and psychological quality in entrepreneurship education (Zhou et al., 2017). The integration of entrepreneurship education into professional courses is of great help to cultivate students’ independent ability (Utami, 2017). Overall, the development of entrepreneurship education for college students in China has started late. Curriculum development and theoretical methods are implemented through the introduction and absorption of foreign excellent experiences. The development of entrepreneurship education in China is still in the exploratory stage.

Firstly, college ideological and political education is introduced alongside college entrepreneurship education, and their contents are studied, respectively, thereby pointing out the differences and connections between them. Innovatively, from the perspective of ideological and political education, the current situation of college entrepreneurship education is explored to effectively implement entrepreneurship education together with ideological and political education. Further, based on the more perfect integrated entrepreneurship education system with ideological and political education, better education and teaching results can be achieved in promoting college students’ entrepreneurial quality and entrepreneurship effectiveness. The purpose is to help college students establish correct ideas and improve the success rate of entrepreneurship. Innovatively, the ideological and political education and entrepreneurship education of college students are combined from the perspective of entrepreneurial behavior of college students. Specifically, the research is conducted through literature review, Questionnaire Survey (QS), and comparative analysis. Afterward, the relationship between college students’ entrepreneurship education and ideological and political education is analyzed based on domestic and international research results, and the role of ideological and political education in college students’ entrepreneurship education is discussed (Jia, 2020). Here, from the perspective of entrepreneurship education, the advantages and disadvantages of current entrepreneurship education are analyzed based on the current situation of ideological and political education and psychological quality of college students, and a QS is designed to collect data for statistical analysis of the effect of ideological and political education on entrepreneurship education. Then, the practical significance of entrepreneurship education is explored, and proposals for improvement are put forward to help ideological and political education better guide college students on the establishment of correct entrepreneurial thinking, improving the success rate of independent entrepreneurship. The results have some reference and guiding significance for higher education institutions’ entrepreneurship education (Hoyos et al., 2017).

## Overview of College Students’ Ideological and Political Education and Entrepreneurship Education

### Review of Domestic and Foreign Research Status

Yang G. (2021) proposed three historical stages of the development of entrepreneurship education in China after investigating and studying the development of entrepreneurship education and demonstrated the development of the expansion stage of entrepreneurship education for college students from 2002 to the current situation. After studying the current situation of entrepreneurship education for college students, Tan (2021) suggested that entrepreneurship education had rich connotations, although there were many problems in the current entrepreneurship education for college students. Meanwhile, he believed that implementing entrepreneurship education was a requirement of socio-economic development, as well as the guarantee of building an innovative country, which had great practical significance and should be vigorously strengthened. After investigation, Yang G. (2021) proposed three current modes of entrepreneurship education and four main implementation approaches and put forward the urgent problems in the current entrepreneurship education for college students. In terms of the relationship between ideological and political education and entrepreneurship education, Fu (2021) argued that the main contents of ideological and political education and entrepreneurship education were intertwined, proving that ideological and political education should always run through the entrepreneurial education system for college students. Research on entrepreneurship education in foreign countries has been carried out for a while, in which some countries have an internationally leading role. These countries put much emphasis on entrepreneurship education in college education. Moreover, they have a mature curriculum system and a faculty with both practical experience and professional knowledge of entrepreneurship, and they have made many achievements in academic research. In terms of the research on the goals and teaching methods of entrepreneurship education in foreign countries, Mozahem (2021) believed that most people thought that entrepreneurship education in universities was primarily to develop students’ innovative thinking, increased their understanding of how new things develop in new fields, and guided students to plan their future career.

### Recent Related Work

In China, the number of college graduates is increasing year by year due to the continuous enrollment expansion, so the employment situation is getting ever-more severe. Thus, it is urgent to improve the entrepreneurial quality of college students, ease the employment pressure, and improve the entrepreneurial education system of college students in China (Fellnhofer and Kraus, 2017). Compared with the Western countries, not much attention has been paid to entrepreneurship education in China because entrepreneurship education has only been introduced in recent decades. In terms of theory and practice, many administrators, teachers, and students in universities still have many shortcomings in their understanding of entrepreneurship education. After years of development, entrepreneurship education for college students has made some achievements, which, however, still has a very big gap compared with the Western countries in improving the entrepreneurial quality of college students and promoting the entrepreneurial effect of college students. As far as ideological and political education is concerned, much theoretical and practical experience has been accumulated in Chinese colleges and universities, and ideological and political education is combined with entrepreneurial education (Zhang and Xu, 2017). Studying entrepreneurship education from the perspective of ideological and political education is of great significance to the smooth development of systematic entrepreneurship education for college students. In May 2010, *The Opinions of the Ministry of Education on Vigorously Promoting the Innovation and Entrepreneurship of College Students in Mountainous Areas* have pointed out to adopt measures to promote college students’ employment. For example, it is necessary to actively encourage college students to start their own businesses. According to the government policies, the Delta region has taken various measures to promote the development of college students in China. At the same time, under the wave of Great Education, many local governments have adopted methods to optimize the social environment of college students. Particularly, preferential policies are formulated to promote college students’ entrepreneurship by reducing taxes, rents, and administrative expenses, as well as by subsidizing enterprise funds and encouraging enterprises to accept college students’ internships, thus further coordinating the relationship between colleges and enterprises in China, which supports college students’ entrepreneurship education. Meanwhile, the role of college entrepreneurship parks is given full play, more colleges and universities are encouraged to establish entrepreneurship parks and provide practical sites for entrepreneurship education. Currently, college education in China is witnessing a golden development stage, which should be fully grasped to promote college students’ entrepreneurship education.

The 18th National Congress report states that “The purpose is to promote entrepreneurship to achieve a higher quality of employment. The proposed employment guidelines include encouraging business startups and making employment a priority for the people. “The report also says, “In the future, we will implement the strategy of giving priority to employment and adopt a more active employment policy. We will promote employment through entrepreneurship, give greater priority to encouraging and supporting entrepreneurship, and do a good job in the employment of young people, especially, college graduates.” In the current education system of colleges and universities in China, entrepreneurship education for college students has not received enough attention. The party and the state always attach importance to the development of ideological and political education in Chinese colleges and universities. In practical teaching of ideological and political education, teachers purposefully and systematically organize or guide students to participate in practical activities according to the teaching objectives and theoretical teaching and help students deepen their theoretical knowledge through the personal experience of ideological and political morality, thereby improving their comprehensive quality (Wang and Chen, 2021). When implementing practical teaching of the college ideological and political courses, teachers should purposefully and systematically transform students’ subjective world through open multilateral interaction and optimize students’ subjective abilities based on the teaching material system, focus on the teaching content, follow the law of Ideological and political work, the law of teaching and educating people, and the law of students’ growth, as well as to the course objectives and requirements (Liu, 2021). Ideological and political education has been developed early in colleges and universities in China and has formed a relatively complete and sound teaching system and teaching mode. Therefore, effective ways to promote the construction of the entrepreneurship education system are studied from the perspective of ideological and political education, which is of great practical significance to realize higher quality college students’ entrepreneurship and employment.

### Overview of College Students’ Ideological and Political Education

As a compulsory university course, ideological and political education includes ideological education, political education, moral education, and legal education, cultivates students with correct and integral ideological and moral characters through social practice, and thereby outputs talents into the society (Chen et al., 2018). In Chinese higher education institutions, Marxism, Mao Zedong Thought, and the theoretical system of socialism with Chinese characteristics are incorporated in the basic theories of ideological and political education, and students’ thoughts and ideology are guided by the Marxist world view, life view, and values view. The fundamental task of ideological and political education is to cultivate a group of the *Four Young* (ideal, moral, cultural, and disciplined youth) for the country.

A large number of excellent socialist successors are urgently needed to better meet the challenges of the new era and cope with the unpredictable international situation. Hence, the importance of ideological and political education has been emphasized by the CPC (Chinese Communist Party) leaders, and the construction of ideological ideology has been underlined again and again. The road of Marxism-Leninism and socialism with Chinese characteristics must be upheld, the patriotic spirit must be inherited and carried forward, and the principle of integration of theory with the practice must be unswervingly implemented. Meanwhile, students should be encouraged to be bold in innovation, mind-liberation, truth-seeking, learning, and progressing, maintaining the advancement and superiority of socialism with Chinese characteristics. With the time marching on, the original mission shouldered on the youth should never be forgotten, and instead, the experience should be summed up for further advancement. In short, more excellent youth should be cultivated for the CPC and the country for the great rejuvenation of the Chinese nation and socialism in China.

Entrepreneurship education is a new educational concept that has emerged with social development and can provide entrepreneurs with skills in technologies, management, and social communication (Xin et al., 2020), help them start businesses in different fields, open up new development paths, inject vitality into social development, and provide more choices for employees. In this way, social development is promoted through entrepreneurs’ self-worth realization. Entrepreneurship education has its origin in the United States (Hassan, 2020), and it aims to cultivate the entrepreneurial ability of college students, thereby moderating unemployment pressure and vitalizing the talents market. With the Chinese economic boom, more and more young people are on their way to entrepreneurship. Yet, quite a few of these new ventures have died prematurely due to insufficient theoretical knowledge, practical experience, and professional guidance. Therefore, scientific and systematic entrepreneurship education should be developed to guide college student entrepreneurs and help them succeed in entrepreneurship (Kirkley and William, 2017).

Entrepreneurship education can help college students improve their entrepreneurial awareness and innovation ability because innovative activities involve practical system engineering, and the demand for integration quality is very high. Thus, entrepreneurship education should enhance the students’ innovative awareness based on solid professional knowledge and skills, cultivate their innovative ability, heighten their social adaptability, and foster their competitiveness and development potential, thereby stimulating industry development vigorously. Through the process, teachers and real entrepreneurs can impart knowledge, thought, spirit, skills, and business specialties to college students to help them determine the appropriate career path (Bischoff et al., 2018).

The content of entrepreneurship education is determined by the goal of entrepreneurship education. The purpose of entrepreneurship education is to cultivate students’ comprehensive ability, especially, the innovative spirit of continuously exploring innovative ideas and development. First, the innovative awareness of college students should be cultivated from many aspects, such as psychology, morality, and moral quality. Second, entrepreneurial knowledge structure, entrepreneurial ability, and strength should be cultivated, which are the practical abilities in entrepreneurship. Specifically, it can be divided into three stages.

**Figure 1:**
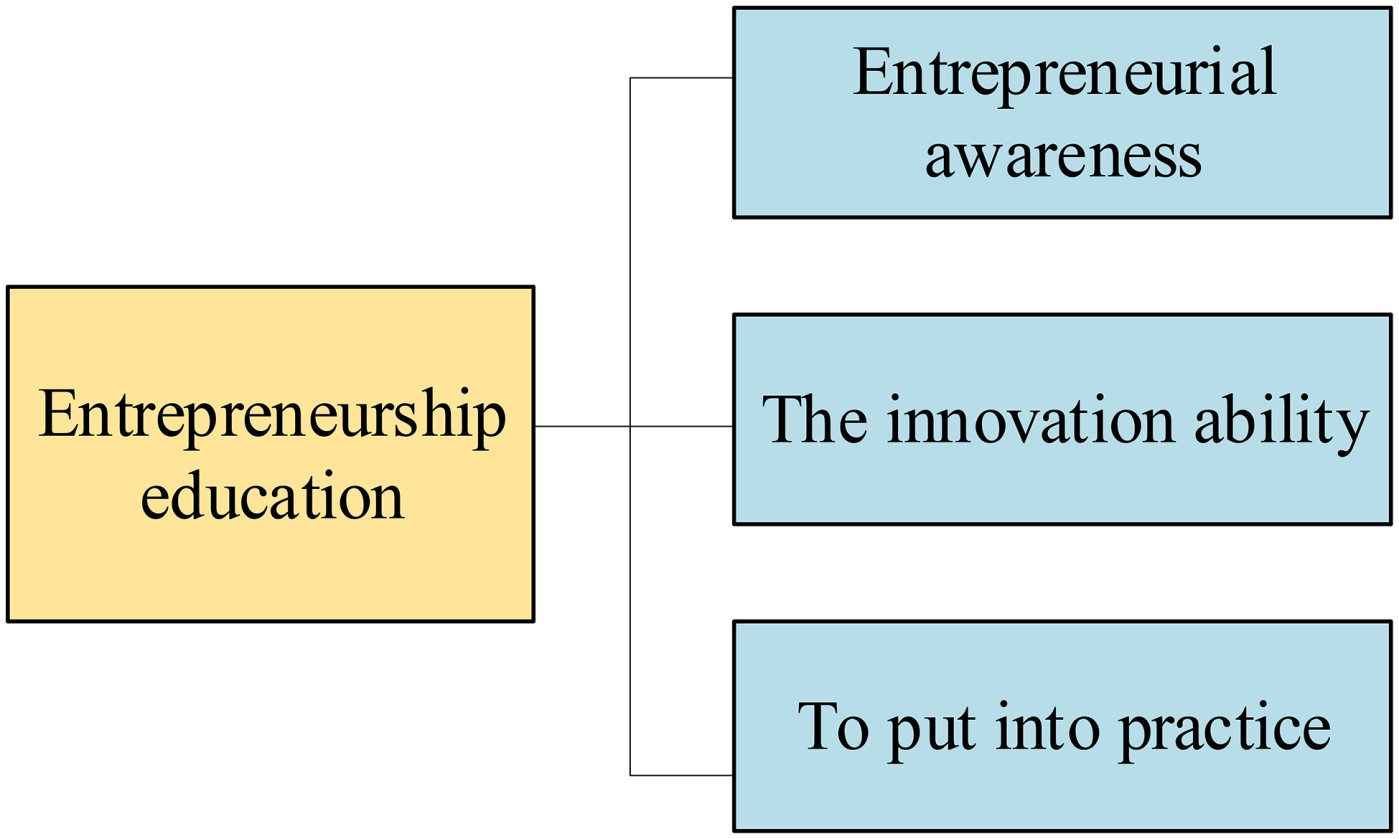
Three stages of entrepreneurship education.

1. In this stage, college students’ entrepreneurial awareness is cultivated. Entrepreneurial awareness refers to the psychological tendency motivated from within, and it is an important part of the entrepreneurial spirit. The entrepreneurial process is driven by many factors, such as creation, interests, and social needs. Entrepreneurial awareness affects the entrepreneurial movement, attitude, and behaviors (Guo, 2020). For entrepreneurs, teamwork ability and communicational skills are essential. Enterprises should be connected to various fields, such as industry, commerce, taxation, and banking, and good relations with customers, business counterparts, and all aspects of the market should be maintained. Therefore, education for the cultivation of social communication ability should be strengthened in innovative industries. Finally, students’ innovative ideas should be cultivated. Innovation is of self-thinking, as well as a practical activity. The innovators deal with a new thing with a new way of thinking through a new way of working (Wang and Jie, 2017).
2. In this stage, students’ innovative knowledge and innovative ability should be cultivated, which is closely related to innovative skills. Firstly, knowledge should be imparted. Solid professional knowledge is the foundation of entrepreneurship. Students should be encouraged to think innovatively, and their entrepreneurial abilities should also be strengthened. Entrepreneurship needs a series of complex and systematic knowledge, including financial management, accounting, production management, logistics management, human resource management, and legal knowledge, such as company law, tax law, property law, and intellectual property law (Gordon and Bursuc, 2018). Innovative energy is the external manifestation of a person’s comprehensive quality and the key factor to determine whether innovative energy can be realized. Many factors are included in innovation. A. The leading force. In most cases, the enterprise is managed by the entrepreneur himself, so he has to evaluate every staff member’s abilities and the living conditions of the enterprise to make the decision and plan strategically. This is an intensive thinking test for the enterprise leader from the external environment. B. Personal ability should be smoothly integrated into team competency to create a favorable and promising working environment, internal and external relations should be fused, and interpersonal relations should be well managed. The motivation of self-learning includes not only the ability to acquire knowledge but also the rapid transformation and application of knowledge. Fourth, innovation is the soul of a nation and an inexhaustible driving force for the prosperity and development of a country. Thus, entrepreneurs should keep pace with the times and innovate from multiple dimensions, including management innovation, technological innovation, and decision-making innovation (Fellnhofer, 2018).
3. In this stage, college students should be correctly guided to put their entrepreneurial thinking, entrepreneurial ability, and entrepreneurial knowledge into practice. The core content of entrepreneurship education is not the teaching of entrepreneurial knowledge and thinking but the transformation of knowledge and thinking into the entrepreneurial ability for successful and independent entrepreneurship. Practices are the only way to find and solve problems in entrepreneurship education (Rusok et al., 2017). Meanwhile, experiences can be accumulated through practice, entrepreneurial awareness and ability can be cultivated, and theoretical knowledge can be transformed into personal experiences. Consequently, entrepreneurs get closer to success step by step through constant attempts and progress (Xu and Xu, 2017). The three stages of entrepreneurship education are shown in Figure 1.

### College Students’ Entrepreneurial Spirit

The entrepreneurial spirit is a spiritual force that guides people to make full use of the existing conditions, give full play to their subjective initiative, pursue opportunities, and create more social value through efforts and innovation. Besides, entrepreneurial spirit is the embodiment of the spirit of the times in employment and entrepreneurial practice, which is manifested in the excellent quality of entrepreneurs and the spiritual style of social organizations. As a powerful spiritual force, it encourages people to innovate and start an innovative career. Particularly, college students’ entrepreneurial spirit refers to the pioneering spirit and innovative thinking ability, as well as diligence, self-reliance, learning spirit, the pursuit of excellence, persistency, and challenging spirit. Thus, the cultivation of entrepreneurial spirit cultivation is an important part of college entrepreneurship education and ideological and political education, which provides a new orientation for higher education reform, during which colleges and universities guide college students to establish a correct concept of employment and entrepreneurship, cultivate students’ awareness of independent career creation, stimulate entrepreneurial spirit, form entrepreneurial quality, continuously improve entrepreneurial quality, and create new value in theoretical and practical learning (Warhuus et al., 2017). The entrepreneurial spirit should be cultivated from both theoretical knowledge and social practices, which should be followed throughout the process of higher education and management and used to guide students to establish self-confidence in learning and work, uphold the sense of responsibility, ideals, and beliefs, give full play to creative thinking, and actively find new opportunities. Thereupon, students can actively acquire and enhance the comprehensive quality for successful entrepreneurship (Li et al., 2020).

### The Relationship Between Ideological and Political Education and Entrepreneurship Education

With the increasing talent competition and mounting college graduates, job hunting is becoming extremely difficult for college students. The college students’ employment path presents a multi-dimensional trend, the employment forms are diverse, and competition among domestic and international talents is getting intense. Analysis of entrepreneurship education shows that it is a systematic process to help students establish the correct concept of entrepreneurship education, which requires time and effort. Entrepreneurship education is the guidance before creative education (Jian, 2017). Entrepreneurship education can help college students form entrepreneurial ideas, shaping them into a fixed innovation machine that stores up strong internal power. As a result, they will be vigorously spurred by the innovation industries and will be encouraged to overcome difficulties and bravely move forward. The entrepreneurial ideas are not innate, and instead, these ideas are inspired through continuous education and cultivation. In ideological and political education, attention is paid to the cultivation of students’ hard-working spirit and innovative ideas, which has laid a good foundation for the college students’ spiritual quality. Students who have been cultivated with the awareness of pioneering and innovation can dynamically cope with employment pressure and try innovation through a self-driven mechanism (Fox et al., 2018). Ideological and political education has a long history in Chinese colleges and universities. In this process, valuable theoretical and practical experience has been accumulated in the contents and methods of ideal education, psychological education, and creationist education, which are the common concerns of entrepreneurship education, thus forming a complete set of teaching systems. However, China’s entrepreneurship education has started at the end of the 20th century with only a few years of history, so there are serious deficiencies in both theory and experience. Importantly, it is necessary to rely on ideological and political education and utilize the advantages of ideological and political education in colleges and universities in terms of experience and teaching methods, which play an important role in promoting the smooth development of entrepreneurship education. At the same time, the development of entrepreneurship education plays a significant role in promoting the reform and innovation of ideological and political education in colleges and universities and improving the effectiveness of ideological and political education.

### The Benefits of Integrating Entrepreneurship Education Into Ideological and Political Education

Innovation ability is a must for every entrepreneur, and it showcases the entrepreneur’s comprehensive quality, including innovative thinking ability, learning ability, leadership ability, communication ability, comprehension ability, and coordination ability. Many educators have realized that disciplines are beginning to be marginalized, and interdisciplinary has appeared, promoting the collaborative progress of different disciplines (Song et al., 2017). Various kinds of knowledge are involved in entrepreneurship, which can no way be covered by entrepreneurship courses. In other words, entrepreneurship education has permeated all disciplines. The ideological and political education is a multidisciplinary course that improves the innovation ability of college students just as innovation ability and vocational ability education. Ideological and political education has great potential in improving college students’ innovation ability.

The cultivation of high-quality talents is the trend of the times and can promote long-term and stable economic growth. Throughout this process, higher education institutions should adhere to the principle of being people-oriented, seeking truth from facts, and keeping pace with the times. The traditional idea of ideological and political education should be inherited with renovation. The educational idea of the new era should be esteemed according to the specific situation and should not be blindingly followed.

Under new ear, innovative talents refer to high-level professional talents with innovative spirit and innovative ability. Cultivating students’ innovative spirit is an important content of ideological and political education, and the cultivation of innovative talents has become an important indicator of ideological and political education in China. For a long time, ideological and political education only passively solves classroom problems for students. To adapt to new changes, the concept of ideological and political education must be revolutionized, the concept of talent training must be updated, the cultivation of professional talents and the cultivation of innovation ability must be set as the new goal of ideological and political education, and students’ innovation ideas an abilities must be transformed into for entrepreneurial activities. Reformations in the traditional teaching method of knowledge inculcation are the premise of social progress.

### QS Design of Entrepreneurship Education for College Students

**Figure 2:**
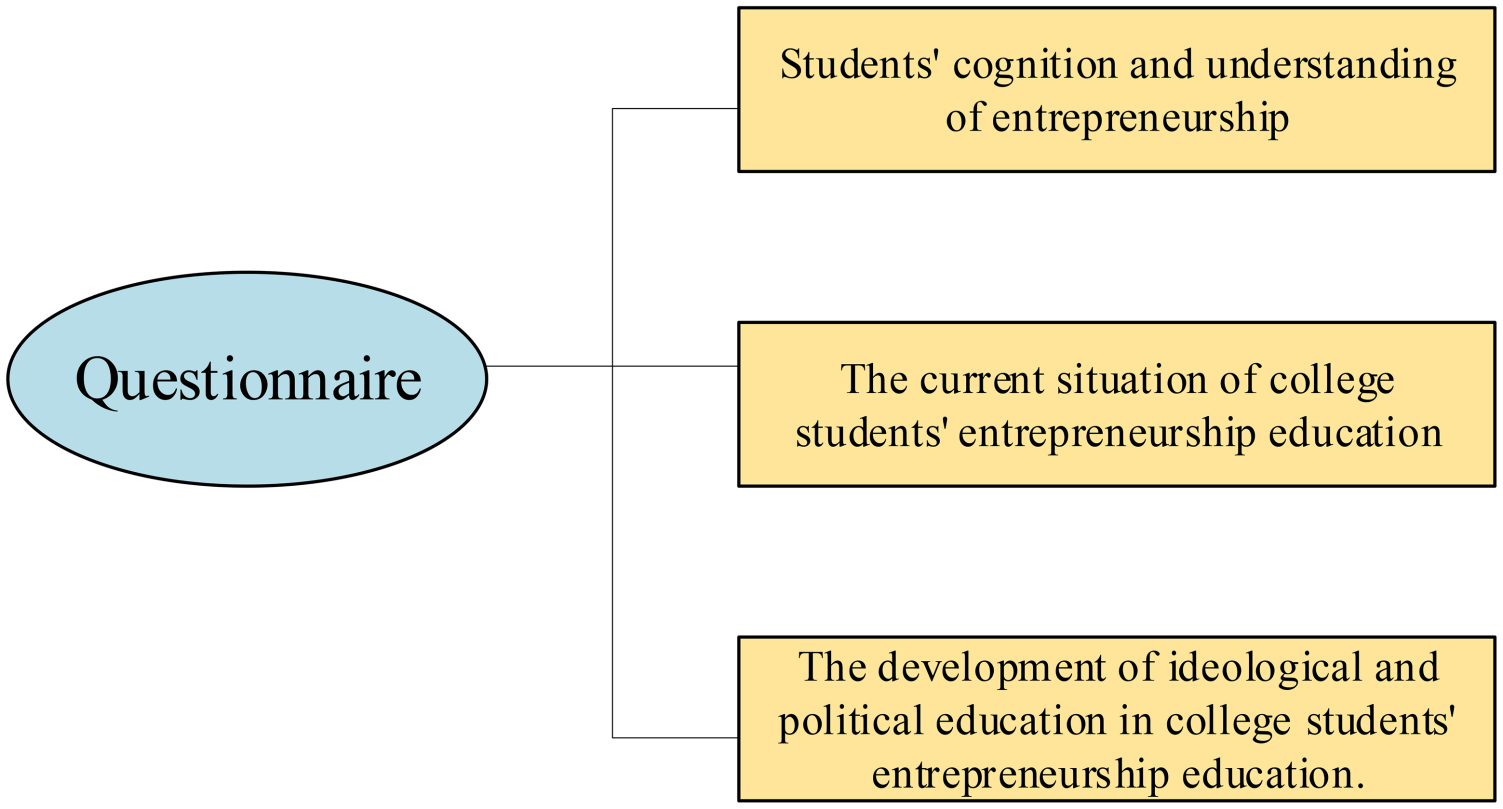
Three parts of the QS.

A QS is designed to investigate the relationship between entrepreneurship education and ideological and political education in higher education institutions and analyze college students’ views on integrating entrepreneurship education into ideological and political education. The QS is divided into three parts, including college students’ cognition and understanding of entrepreneurship, the current situation of college students’ entrepreneurship education, and the development of ideological and political education in college students’ entrepreneurship education. The structure is shown in Figure 2.

The items of this QS are set by objective questions and supplemented by subjective questions. The QS is distributed in three local universities, and the respondents include all college students from freshmen to seniors. The QS is distributed on the principle of voluntary participation. Here, the QS is issued to three local higher education institutions. The contents of the QS focus on college students’ views, suggestions, and recognition of ideological and political education, a total of 300 QSs are distributed, and 288 valid QSs are collected, with a recovery rate of 96%. The subjects are college students. Viewing the number of participants, this QS adopts the combination of stratified sampling and random sampling, as well as qualitative and quantitative analysis to arrange time scientifically and reasonably. Consequently, an in-depth understanding of the current college students’ cognition and understanding of entrepreneurship can be obtained, along with the current situation of college students’ entrepreneurship education and the development of ideological and political education in college students’ entrepreneurship education by analyzing the meaning behind the statistical data (Chen, 2019). The random sampling method is adopted to ensure the fairness of the QS results. The questions in the QS are shown in Table 1.

**Table 1:** The distribution of key items in the QS of college students’ entrepreneurship education and the questions settings.

Questions	Options
Are you interested in entrepreneurship education?	A: Very interested;
	B: Interested;
	C: General;
	D: Not so interested; E: No interested at all;
	A: Three or more times a week;
How often do you receive entrepreneurship education in school?	B: Twice a week;
	C: Once a week;
	D: Once a month or less;
	E: Never have received;
	A: Absolutely necessary;
Do you think it is necessary to set up systematic entrepreneurship education in university?	B: Necessary;
	C: Indifferent;
	D: Not necessary at all;
	A: Very effective;
Do you think entrepreneurship education is effective in improving your ability?	B: Effective;
	C: General;
	D: Not effective at all;

### Reliability and Validity Test of QS

In essence, QS is a problem set used for sorting, analyzing, and evaluating data scientifically collected from a specific socio-economic phenomenon. Importantly, the reliability and validity of the QS should be analyzed to ensure the accuracy and scientificity of the survey results. QS reliability refers to the degree of internal consistency and external stability of the survey results. First, the reliability should be analyzed: After the collected data are sorted out, the invalid QSs are eliminated, as well as the questions with factor load less than 0.6, and the questionnaire data are analyzed through the confirmatory analysis method. The results show that the overall internal consistency reliability of the scale is 0.89, and the α coefficient of the sub-dimension is between 0.67 and 0.81. In terms of content validity, each item of the scale is the result of collating open QS of college students and is consistent with the content of relevant literature, individual interviews of college students, and expert surveys. In terms of structural validity, the correlation analysis method is used to test the structural validity of the scale, that is, the validity of the scale is estimated by the correlation between various factors in the scale and the correlation between each factor and the total score of the scale. The results show that the correlations between different dimensions and the total score range from 0.50 to 0.83, and the correlations between dimensions range from 0.35 to 0.64, which are in line with the statistical indicators of the scale.

## Analysis of QS Results

### QS of College Students’ Interest in Entrepreneurship Education

**Figure 3:**
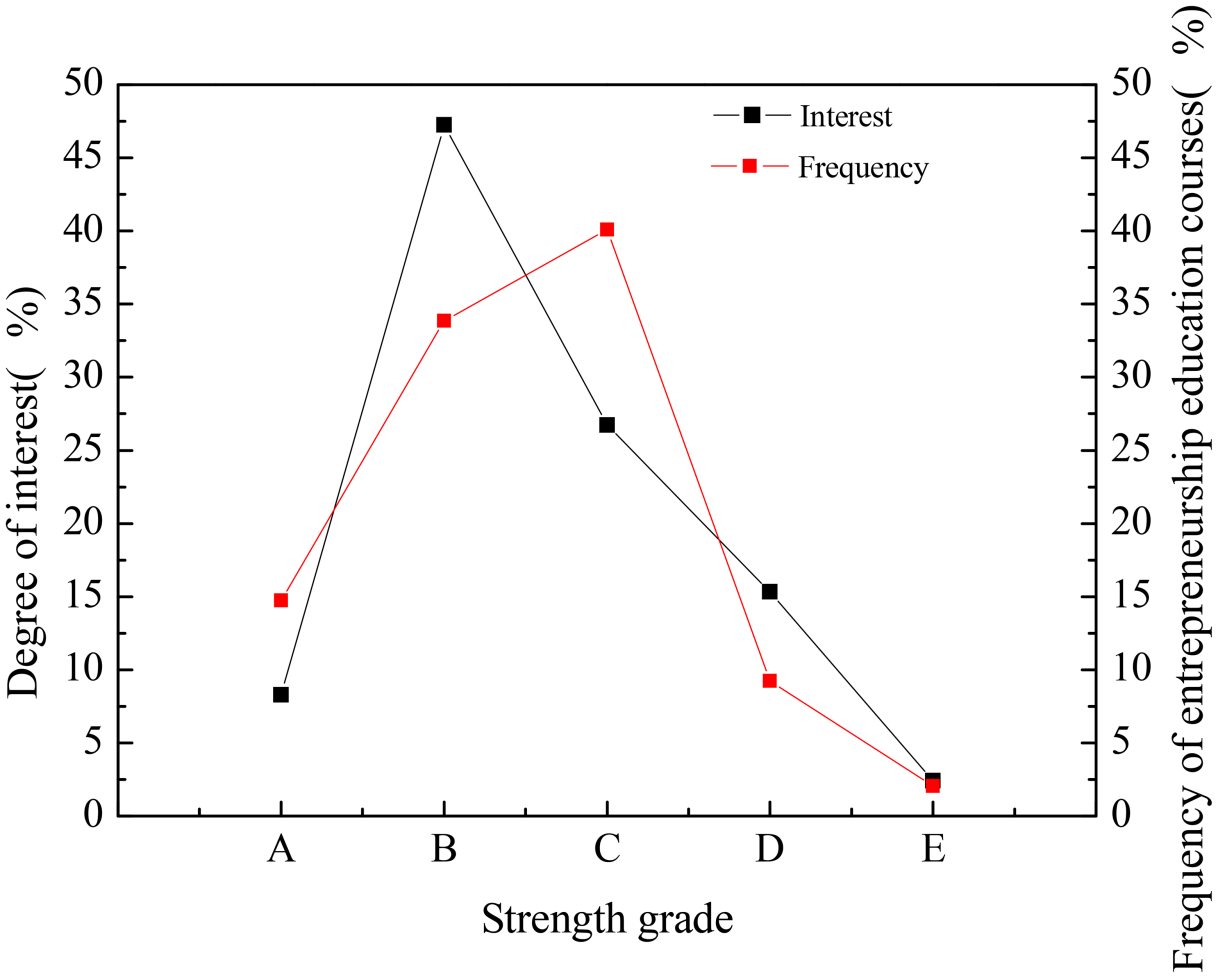
Statistical results of the interest of college students in entrepreneurship education and the frequency of receiving entrepreneurship education.

The QS is sorted out, and the comparison of college students’ interest in entrepreneurship education and the frequency of receiving entrepreneurship education is shown in Figure 3.

In Figure 2, the strength grade of the horizontal coordinate decreases successively from A to E, A means that students are very interested in, or they often receive entrepreneurship education, while E represents that they are not interested in, or they have never received entrepreneurship education at all. The results suggest that the current understanding of entrepreneurship education needs to be deepened. Only less than 10% of the students are very interested in entrepreneurship education. Most students are only interested in entrepreneurship education; in other words, they are only being curious. The students who are in the general interest, not so interested, and not interested accounts for 26.72, 15.34, and 8.27%, respectively. Meanwhile, the group of students who show higher interest degrees has received more frequent entrepreneurship education, and students’ interest in entrepreneurship education and frequency of receiving entrepreneurship education are positively correlated. It indicates that entrepreneurship education in colleges and universities has not been popularized. Besides, most students have a wait-and-see attitude toward entrepreneurship education and do not realize the importance of entrepreneurship education. Part of the reason is that college students’ general enthusiasm for entrepreneurship is not high. In short, entrepreneurship education is dispensable for most college students, they have a relatively low entrepreneurial intention, they believe mastery of professional knowledge is not enough to tackle the severe employment situation, and their self-recognition is very limited.

### Entrepreneurship Education in the Ideological and Political Class

**Figure 4:**
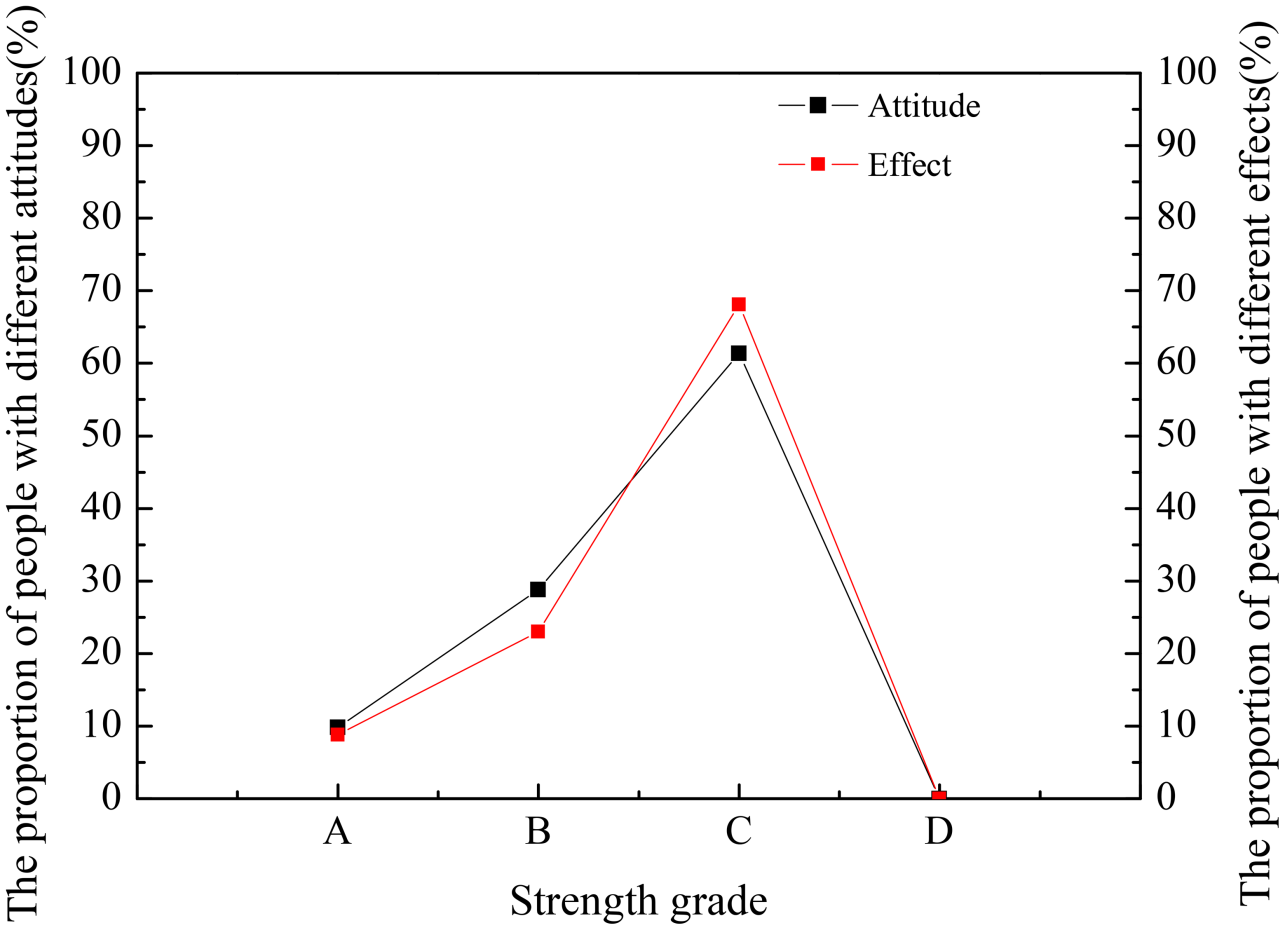
Comparison of students’ attitudes and effects of entrepreneurship education in ideological and political classes.

Students’ attitudes and the effect of entrepreneurship education in the ideological and political classroom are compared and statistically analyzed in Figure 4.

**Figure 5:**
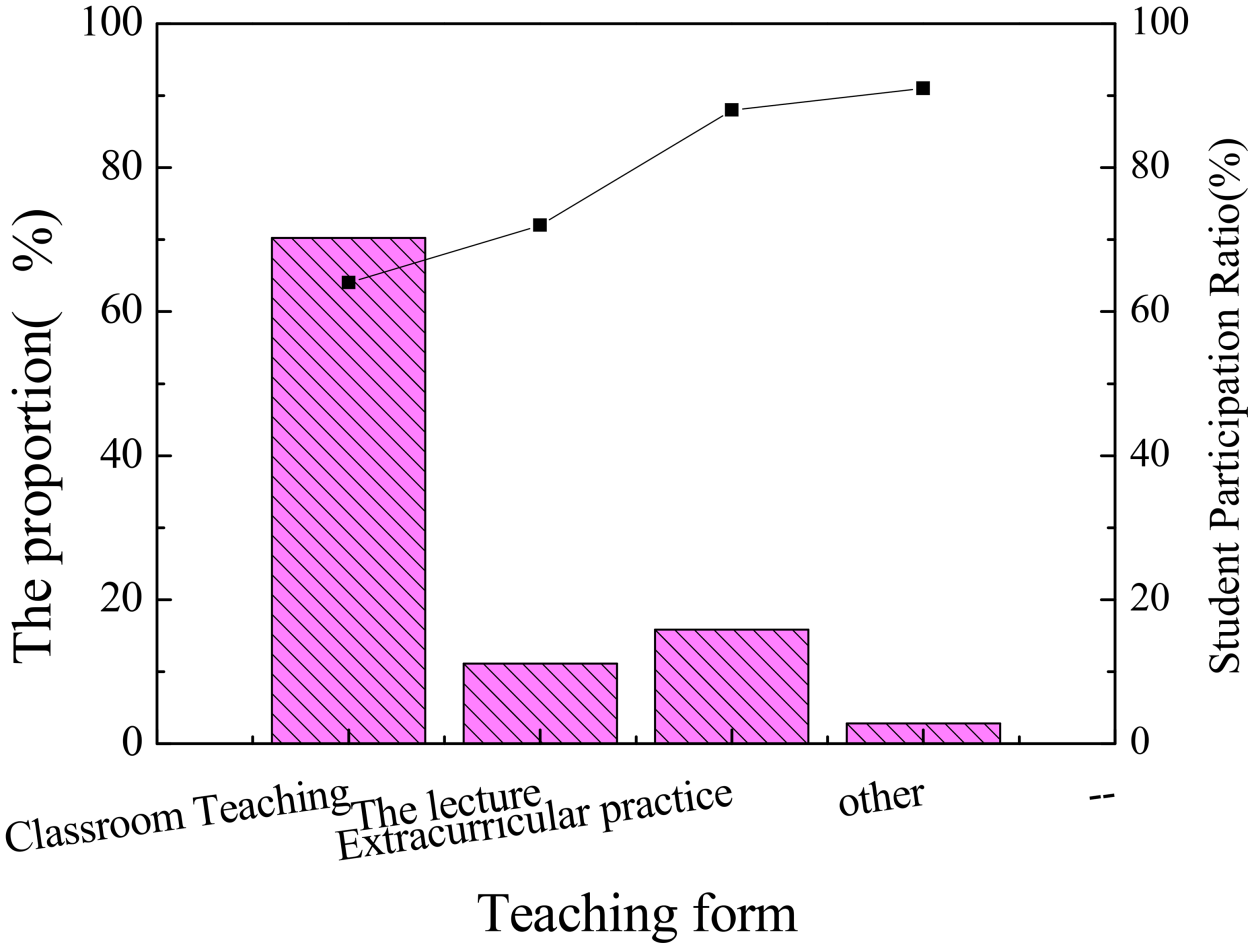
Statistics of main teaching patterns and students’ participation in entrepreneurship education.

In Figure 3, A, B, C, and D represent four successively decreasing levels, respectively. Specifically, A represents that entrepreneurship education is very effective, while D represents not effective at all. Figure 3 implies that the integration of entrepreneurship education into the ideological and political class has no great effect on most students. Only about 10% of the students believe that entrepreneurship education has great help to the growth and development of individuals, and few students think entrepreneurship education is very effective. Most students are indifferent toward entrepreneurship education. Due to the sophisticated psychological effect, they subjectively believe that the development of entrepreneurship education has no practical effect on their career planning. Most possibly, the content of entrepreneurship education in ideological and political education is too little. Moreover, the main forms of entrepreneurship education are theoretical education, and without sufficient practical activities, entrepreneurship education cannot exert its role sufficiently in students’ entrepreneurship. As a result, most students believe that the role of entrepreneurship education for their growth is very limited. Currently, the teaching forms of entrepreneurship education in the ideological and political classroom include classroom teaching, lectures, extracurricular practice, and other forms. The proportion of different forms is shown in Figure 5.

Figure 4 illustrates that entrepreneurship education is mainly carried out through classroom teaching, while extracurricular practice only accounts for 15% of total courses. In entrepreneurship education, the need to practice is much more than theoretical education, and the teaching effect is greatly reduced by armchair theories. Most students do not think highly of entrepreneurship education because the ideological and political education of the higher education institutions has not been successful in the combination of theories and practice and has not given full play to the role of entrepreneurship education and ideological and political education. The effective integration of the multidiscipline relies on the characteristics of ideological and political education, such as inclusiveness and thinking orientation through imperceptible cultivation of innovative ability and innovative thinking. This will surely be the direction for entrepreneurship education. In conclusion, the main form of ideological and political education should not be confined within the classroom, and the theory should be well-combined with practice, so that ideological and political education can play the utmost role.

### The Situation of Entrepreneurship Education Under University Teachers

**Figure 6:**
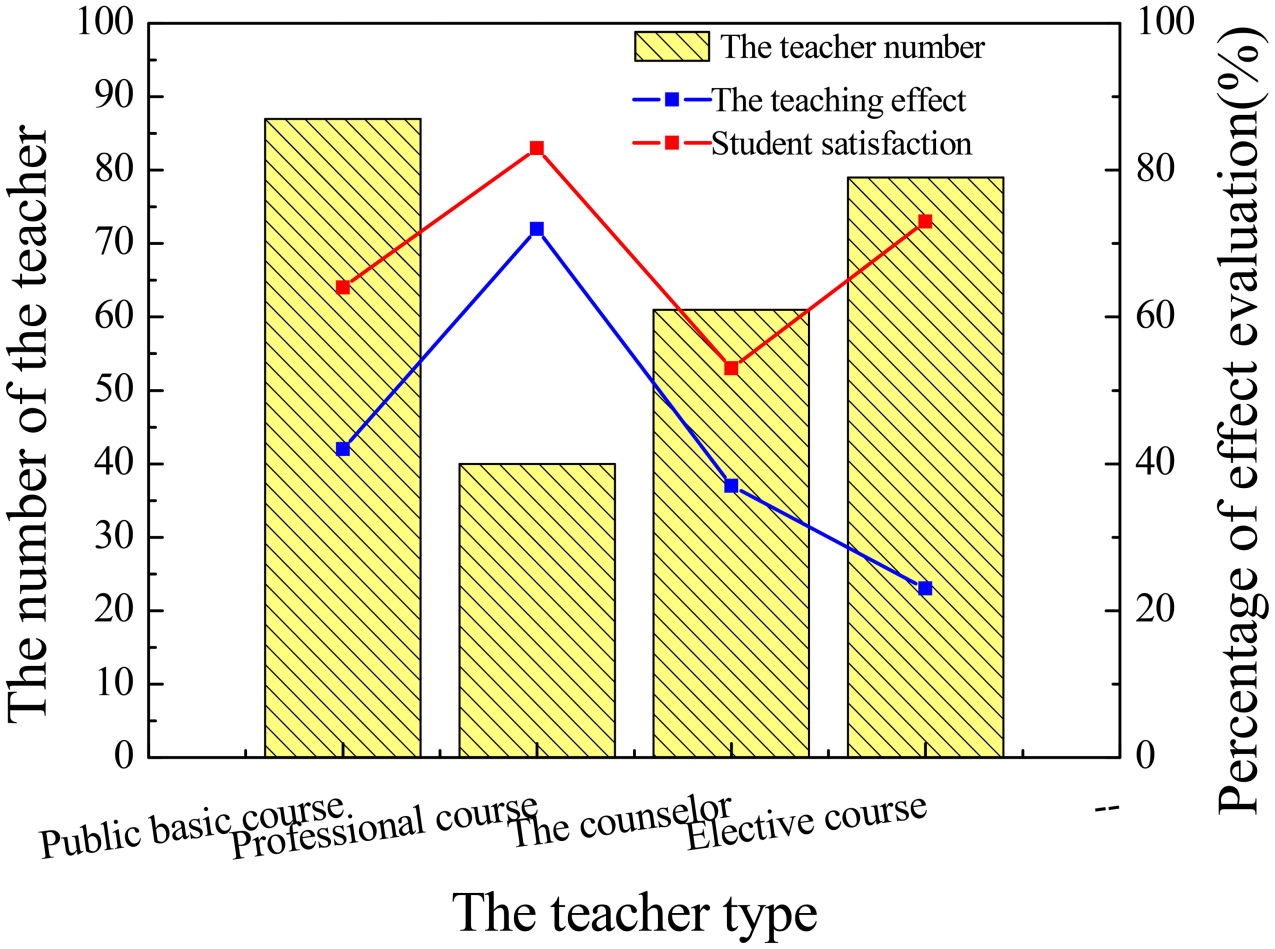
Comparison of the number of teachers offering entrepreneurship education and the proportion of excellent teaching effect evaluation.

Figure 6 displays the statistical results of different types of university teachers who are offering entrepreneurship education and the teaching effect.

Figure 5 displays that most entrepreneurship education teachers are from public course teachers, and the second most groups are from elective teachers. Counselors who comprehensively guide students’ learning and life also teach entrepreneurship education from time to time, while professional entrepreneurship education teachers account for the least in all teaching staff. According to the students’ evaluation of teachers’ impact, more than 70% of students believe that teachers of professional courses have a great impact on them. Meanwhile, according to the QS results of students’ satisfaction with teachers’ teaching content, the satisfaction with teachers of professional courses is more than 80%, while the satisfaction with teachers and counselors of public courses is relatively low. However, according to the QS results, the professional teachers’ entrepreneurship education is the most helpful to students, which can be attributed to the fact that the knowledge and skills of the professional teachers help improve students’ entrepreneurial ability. Therefore, the number of professional teachers in entrepreneurship education should be increased.

## Results and Discussion

According to the overall results of the QS, the goal of ideological and political education in entrepreneurship education for college students is not clear, which directly causes the lack of pertinacity of entrepreneurship education content for college students. The ideological and political education in college students’ entrepreneurship education should keep pace with the development of times, and it should encourage their entrepreneurial awareness, innovative spirit, and entrepreneurial ability based on the actual needs of college students, thereby cultivating comprehensive entrepreneurial talents with sound entrepreneurial character and all-around development. So far, most Chinese colleges and universities focus more on the cultivation of entrepreneurial skills and lack a real-time and comprehensive analysis of the market entrepreneurial environment. Thus, the content of ideological and political education in college students’ entrepreneurship education is not targeted.

Additionally, the ideological and political education content of entrepreneurship education in colleges and universities has not combined the current entrepreneurial psychological state and entrepreneurial thought of college students. According to statistics, 53.2 and 58.9% of college students who do not want to try to start a business, for the time being, are afraid of the pressure that comes with entrepreneurship. Many cannot afford setbacks and the hardship on the road to entrepreneurship, so they choose to give up. This psychological state determines the attitude and view of college students toward entrepreneurship. Among the students who want to start their businesses, more than 31% of them start their businesses out of desperation to cope with the current severe employment situation, and their entrepreneurial motivation is very passive, while 43.6% of them think entrepreneurship means being a boss and getting rid of constraints. Under such motivations, students yearn for quick success and quick benefits when starting a business, and once they encounter setbacks and unfavorable circumstances during entrepreneurship, they may easily get confused. Besides, other survey shows that more than 80% of students are not satisfied with the current entrepreneurship education in schools and believe that the entrepreneurship education only focuses on the theoretical knowledge and does not analyze practical business cases. At the same time, under the survey of students’ expectations for the future development of entrepreneurship education, more than 80% of students hope that the school can establish an entrepreneurship practice base and give them full opportunities to practice. It shows that college students have an urgent need to improve the practical teaching system of entrepreneurship education. The content of ideological and political education in entrepreneurship education should be better reflected in practical teaching to provide help and guidance for college students to start their businesses.

## Conclusion

Here, a brief introduction is given to the concept and development of entrepreneurship education and ideological and political education, and the integration of entrepreneurship education into ideological and political education is proposed. So far, few people have studied the development of entrepreneurship education from the perspective of ideological and political education. Here, innovatively, the popularization of ideological and political education is also explored through the entrepreneurship education of college students. Specifically, from the ideological and political perspective, the problems and causes of the development of entrepreneurship education are analyzed through the QS method and statistical data analysis. Finally, feasible ways to build the entrepreneurial education system for college students are promoted by strengthening ideological and political education. The internal favorable factors and external conditions are analyzed for ideological and political education. Subsequently, a QS is conducted on three local higher education institutions, finding that college students have an incomplete understanding of entrepreneurship education. The inclusiveness and thinking guidance role of ideological and political education must be exerted to infiltrate entrepreneurship education into students’ thinking. There are still some shortcomings. The scope of the QS is limited to the local higher education institutions, so the results are not comprehensive and cannot evaluate the overall situation of all higher education institutions in China. Due to limited theoretical knowledge, the views may get somewhat biased. Thus, the research results may be not comprehensive enough to get popularize the combination of ideological and political education with entrepreneurship education for college students. Additionally, the exploration of ways to improve ideological and political education and promote entrepreneurship education should be further improved. At the same time, the scope of the investigation is narrow, mainly concentrated in three universities, so the understanding of the actual situation of entrepreneurship education in colleges and universities is not specific enough, and the analysis is not comprehensive enough. The QS is only distributed to local colleges and universities, so the results are not comprehensive and cannot be used to evaluate the overall situation of all the Chinese colleges and universities. In the follow-up research, it is hoped that the scope and size of the QS will be expanded. It is self-evident that with the progression of teaching content and mode, entrepreneurship education will be gradually accepted by more college students. Currently, college students’ entrepreneurship education has received insufficient attention in China, while emphasis has always been on the development of ideological and political education, which has been developed before the popularization of college education and has formed a relatively complete and desirable teaching system. Hence, here, the practical significance of promoting the entrepreneurship education system through the combination of ideological and political education is explored to cultivate more high-quality college students and promote their employment.

## Data Availability Statement

The raw data supporting the conclusions of this article will be made available by the authors, without undue reservation.

## Ethics Statement

The studies involving human participants were reviewed and approved by Northwestern Polytechnical University Ethics Committee. The patients/participants provided their written informed consent to participate in this study. Written informed consent was obtained from the individual(s) for the publication of any potentially identifiable images or data included in this article.

## Author Contributions

The author confirms being the sole contributor of this work and has approved it for publication.

## Conflict of Interest

The author declares that the research was conducted in the absence of any commercial or financial relationships that could be construed as a potential conflict of interest.

## Publisher’s Note

All claims expressed in this article are solely those of the authors and do not necessarily represent those of their affiliated organizations, or those of the publisher, the editors and the reviewers. Any product that may be evaluated in this article, or claim that may be made by its manufacturer, is not guaranteed or endorsed by the publisher.
